# Responses of photosynthetic characteristics of oat flag leaf and spike to drought stress

**DOI:** 10.3389/fpls.2022.917528

**Published:** 2022-07-28

**Authors:** Haoqi Tian, Qingping Zhou, Wenhui Liu, Jing Zhang, Youjun Chen, Zhifeng Jia, Yuqiao Shao, Hui Wang

**Affiliations:** ^1^Sichuan Zoige Alpine Wetland Ecosystem National Observation and Research Station, Southwest Minzu University, Chengdu, China; ^2^Academy of Animal Science and Veterinary Medicine of Qinghai Province, Xining, China; ^3^Sichuan Animal Science Academy, Chengdu, China

**Keywords:** non-leaf organ, C_4_ pathway, relative water content, oat, glume

## Abstract

Raising crops production *via* improving photosynthesis has always been focused. Recently excavating and increasing the photosynthetic capacity of non-leaf organs becomes an important approach to crops yield increase. Here we studied the photosynthetic characteristics of the flag leaf and the non-leaf organs including the sheath, the glume and the lemma under greenhouse. The relative water content (RWC), the stomatal characteristics, the photosynthetic pigment contents, the enzyme activities in C_3_ and C_4_ pathway and the malate content of the flag leaf and the non-leaf organs on 7, 14, 21, and 28 days after anthesis (denoted by 7DAA, 14DAA, 21DAA, and 28DAA) were determined under well-watered (CK) and water-stressed (D) treatments. Drought stress significantly reduced the RWC of the flag leaf and the non-leaf organs, while the variation of RWC in the glume and the lemma was lower than in the flag leaf. The chlorophyll a content, the chlorophyll b content, the total chlorophyll content and the xanthophyll content in the flag leaf were significantly decreased under D. However, drought stress significantly increased the photosynthetic pigment contents in the glume at the late stage (21DAA and 28DAA). In addition, the induced activities of PEPC, NADP-MDH, NADP-ME, NAD-ME, and PPDK in non-leaf organs under drought stress suggested that the C_4_ photosynthetic pathway in non-leaf organs compensated the limited C_3_ photosynthesis in the flag leaf. Non-leaf organs, in particular the glume, showed the crucial function in maintaining the stable photosynthetic performance of oat.

## Introduction

Oat is an important crop ranking around sixth in the cereal cultivated area (FAO, 2019), versatilely utilized as grains and forage. Oat grain contains high levels of β-glucan and dietary fiber components and is a source of food, pharmaceutical and industrial products (Zaheri and Bahraminejad, 2012; Marshall et al., 2013; Gorash et al., 2017). In China, oat is grown over a wider area, mainly in the north and southwest, with an annual harvested area of 0.7 million ha, yielding 8.5 million tons (Diao, 2017; Zhou et al., 2018). The regions cultivating oat in China is characterized by arid and semiarid climate especially in northwest (Li et al., 2019), where water deficit is one of the major constraints for the growth and production of oats (Stevens et al., 2004; Hakala et al., 2020). Drought caused oat grains yield loss of 32%–69% (Varga et al., 2013; Zhao et al., 2021), which was associated with a marked reduction in photosynthesis ability under drought stress (Marcińska et al., 2017). Over 90% of grain yield originated from photosynthetic production, and traditionally, the flag leaf acted as the main assimilation organ for the cereal crops (Evans et al., 1980). Nevertheless, other organs, such as the reproductive structures, were often considered to be the carbon sinks, while were shown to be photosynthetically active (Hu et al., 2014; Sanchez-Bragado et al., 2014; Wang et al., 2020). Recently ear photosynthesis has been focused on its prominent compensation, especially subjected to water deficit (Tambussi et al., 2005; Abebe et al., 2010; Hein et al., 2016; Lou et al., 2018). The reported ear photosynthetic contribution to grain filling ranged from 12% to 65% (Maydup et al., 2010; Sanchez-Bragado et al., 2014; Hu et al., 2019) and the proportion was enhanced with the decrease of water supply (Zhang et al., 2011).

Drought stress inhibited all stages of plant growth and development, especially during the reproductive stage. When subjected to water deficit, the leaves would wilt and senesce due to the water loss (Abebe et al., 2010). Nevertheless, the ear of cereal crops was more resilient, maintaining the stable moisture state under drought stress (Tambussi et al., 2005; Li et al., 2017). In comparison with the leaves, a higher RWC (relative water content) of the non-leaf organs was reported in wheat (Wardlaw, 2002; Tambussi et al., 2005), barley (S’anchez-D’ıaz et al., 2002) and cotton (Hu et al., 2014), which could be explained by the higher osmotic adjustment and the xeromorphic anatomy. The osmotic adjustment was substantially higher in non-leaf organs than in leaves (Tambussi et al., 2005; Hein et al., 2016). In addition, the unique anatomical characteristics, including the thicker wax layer, smaller intercellular spaces and thicker cells, contributed to the higher water use efficiency in the non-leaf organs than in leaves (Blum, 1985; Li et al., 2006). The better photosynthetic performance of non-leaf organs was associated with the ability to maintain a steadier water state under drought stress conditions.

Apart from the higher ability to adjust water state under drought stress, the higher photosynthetic efficiency pathways were considered to sustain grain-filling in the non-leaf organs of C_3_ plants. A large body of evidence indicated C_4_ pathway, C_4_-like pathway or C_3_-C_4_ intermediate pathway might conduct photosynthesis in non-leaf organs of wheat (Ziegler-Jöns, 1989; Rangan et al., 2016; Balaur et al., 2018), barley (Nutbeam and Duffus, 1976), cucumber (Sui et al., 2017), tobacco and celery (Hibberd and Quick, 2002). Baluar et al. (2018) observed that similar to maize leaf, the glume, the lemma and the awn had the Kranz anatomy with two types of chloroplasts in wheat. In addition, the isotope labeling experiment indicated that most of ^14^C was detected in malate after assimilating ^14^CO_2_ by the illuminated ear of wheat (Singal et al., 1986). Some important photosynthetic enzymes in the C_4_ pathway exhibited activities in non-leaf organs of C_3_ plants. The key enzyme, PEPC, was determined with higher activity in non-leaf organs than in leaves and the activity of PEPC increased under drought stress conditions (Hu et al., 2014; Jia et al., 2015; Wang et al., 2020). Previous studies also reported that photosynthetic enzymes involved in three classical C_4_ photosynthesis subtypes, NADP-ME, NAD-ME, and PEPCK, conducted carbon fixation in the non-leaf organs (Singal et al., 1986; Wei et al., 2003; Zhang, 2019). Besides the anatomical and zymologic evidences, the molecular evidence verified that a complete set of C_4_ specific genes including *ppc*, *aat*, *mdh*, *me2*, *gpt*, and *ppdk* were up-regulated in caryopsis and NAD-ME type C_4_ photosynthesis operated in developing wheat grains (Rangan et al., 2016). The C_4_ pathway originated later than the C_3_ pathway, while provided enhanced radiation-water-and nitrogen-use efficiency especially in sub-optimal environments (Paulus et al., 2013). Thus, investigating and utilizing the C_4_ photosynthetic ability in the non-leaf organs of C_3_ plants attracted the attention of plant physiologists and crop breeders.

## Materials and methods

### Experimental design

*Avena sativa* cv. Junma seeds were sown in the plastic pots (height 25 cm, diameter 27 cm) in the greenhouse of the Academy of Animal Science and Veterinary Medicine of Qinghai Province in June 2020. Each pot was filled with the field soil (weight 5 kg, maximum field capacity 35.44%) from Huangzhong County in Qinghai Province and 24 pots were used in this study. The field soil was mixed with 0.3 g urea (containing 46% N) and 0.3 g diammonium phosphate (containing 18% N and 46% P_2_O_5_) per kg soil. Before sowing, 2 L water was added to each pot. When the height of seedlings was around 10 cm, six plants were reserved in each pot. All plants were well-watered (75% of maximum field capacity, CK) till 7 days after anthesis, when half of the pots were started to water with 45% of maximum field capacity, denoted by D. CK and D treatments were controlled till seed maturation by weighing and watering the pot by using an electronic scale at 6:00 p.m. each day. The flag leaf, the sheath, the glume, and the lemma in the plant were collected at 7, 14, 21, and 28 days after anthesis, denoted by 7DAA, 14DAA, 21DAA, and 28DAA, under CK and D treatments with three replicates. All samples were saved in the refrigerator with −80°C for measuring the physiological parameters.

### Relative water content

The flag leaf, the sheath, the glume, and the lemma samples were collected, immediately weighed to obtain the fresh weight (w1) and soaked into the distilled water for 30 h. After being weighed again, thus obtaining the saturated weight (w2), the samples were dried in an oven until constant weight (w3). The relative water content (RWC) was calculated based on the following formula.


RWC(%)=((w1−w3)/(w2−w3))×100.

### Stomata characteristics

The adaxial and abaxial surfaces of the leaf, the interior and exterior surfaces of the sheath, the glume and the exterior surfaces of the lemma on 7DAA were wiped with wet paper. The clear nail polish was smeared on both sides of four organs from 10:00 a.m. to 11:30 a.m. After 20 min, the dried nail polish was peeled away and pressed against the glass slide. The samples were observed under a microscope (DM2000 LED, Leica, Germany) at 40× magnification and the sight area was 272.03 μm × 207.61 μm. From each sample, 10 random sights were selected to record cell numbers, stoma numbers and guard cell length. Stomatal frequency and stomatal index were calculated by the following formula.


$$\begin{array}{c} \mathrm{Stomatal index}=\left(\mathrm{stoma number}/\left(\mathrm{stoma number}+\mathrm{cell number}\right)\right)\\ \times 100\% \end{array}$$

Stomatal frequency=stoma number/sight area

### Photosynthetic pigment content

The flag leaf, the sheath, the glume, and the lemma samples were snipped into small strips and transferred to the centrifuge tubes with 10 ml of extracting solution. The extracting solution was mixed with acetone and absolute ethyl alcohol by the volume rate of 1:1. Absorbancy of extracting solution at 470, 663, and 645 nm was measured by using the full wavelength microplate analyzer (Multiskan GO, Thermo Fisher, United States). The chlorophyll a content (chla), the chlorophyll b content (chlb), the total chlorophyll content (total chl) and the xanthophyll content (xan) were calculated by the following formula.


chla(mg/g)=((12.7×D663−2.69×D645)×V)/(1000×W)

chla(mg/g)=((22.9×D645−4.68×D663)×V)/(1000×W)

totalchl(mg/g)=chla+chlb

$$\begin{array}{c} \mathrm{xan}\left(\mathrm{mg}/g\right)=\left(1000\times D470-3.27\times \mathrm{chla}-104\times \mathrm{chlb}\right)\\ /\left(229\times V\right)\times \left(1000\times W\right)) \end{array}$$

where, V is the volume of extracting solution, W is the weight of the sample.

### Photosynthetic parameters

Pn (net photosynthetic rate) and Gs (stomatal conductance) of the flag leaf, the sheath, the glume, and the lemma were determined by using a portable photosynthesizer (Li-6800, Li-Cor, United States) at 9:00 a.m.–11:30 a.m. We chose the gasket of air chamber with the smallest size of 2 cm^2^. The 3 × 3 light source provided independent control of red and blue light intensities.

### Photosynthetic enzyme activities

The samples saved in the −80°C refrigerator were used to determine the activities of Rubisco, PEPC, PPDK, NAD-ME, NADP-ME, and NADP-MDH. A 0.1 g sample was ground using a mortar at 4°C and then the grinding media of 1 ml was added. Samples were centrifuged at 8,000 *g* for 10 min at 4°C. The supernatant was used for the activities assays. The grinding media and the reaction mixture solutions were assay kits from the company of Suzhou Comin Biotechnology Co., Ltd. The absorbancy was recorded at 20 s and 5 min 20 s from the starting, respectively, at the wavelength of 340 nm using the full wavelength microplate analyzer (Multiskan GO, Thermo Fisher, United States).

### Malate content

An 0.1 g of sample was immediately frozen in liquid N_2_, ground thoroughly and soaked in the centrifuge tube with 1 ml of distilled water. The tube was placed in the 4°C refrigerator overnight. Following centrifugation at 8,000 *g* for 10 min at 4°C, the supernatant was filtered with the needle-type filter. The malate content was measured using an HPLC system (High Performance Liquid Chromatography, L3000, RIGOL, China) containing a Rigol C18 reversed-phase column (250 nm × 4.6 nm, 5 μm). The mobile phase was 0.016 M NaH_2_PO_4_ (pH 4.0) with a flow rate of 0.8 ml/min. The column temperature and the sampling time was 25°C and 30 min, respectively. The injection volume was 10 μl. Samples were detected at 214 nm.

### Quantitative real-time PCR (qRT-PCR) analysis

Total RNA was extracted from the samples using the Pure Plant Total RNA Extraction Kit (TSINGKE, Beijing) as described in the manufacturer’s instructions. The ratio of absorbancy at 260–280 nm was measured by Nanophotometer of Implen (Implen, Germany). RNA integrity was detected by agarose gel electrophoresis. All samples were stored at-80°C. All RNA samples served as templates for the cDNA synthesis by the Goldenstar RT6 cDNA Synthesis Mix(TSINGKE, Beijing). The reverse transcribed products was kept at −20°C.

Quantitative real-time PCR (qRT-PCR) was performed with Step One Plus real-time quantitative PCR instrument (Thermo Fisher, America) and using the SYBR Green I PCR master mix kit (TSINGKE, Beijing) according to the Cmanufacturer’s instructions. The relative amount of gene expression was calculated using the expression of actin as internal control gene. qRT-PCR primers were as following (Table 1). The relative quantity of gene expression was calculated using 2^–ΔΔCT^ method (Livak and Schmittgen, 2001).

**Table 1:** Sequence of primers used for qRT-PCR.

Primer name	Forward 5′–3′	Reverse 5′–3′
*AS-PEPC*	TGCGGTTGCGTGAGTCATACATC	TCAGCAGGCTCCTTCTCATCGG
*AS-MDH*	AACCACTCGTCCAGTCAGTACCC	CGCATTGAGCCATTCATCGTCTTG
*AS-ACT*	AGCTCGCATATGTGGCTCTTGACT	TCTCATGGATTCCAGCAGCTTCCA

AS, *Avena sativa*.

### Statistical analysis

The Relative water content, the stomata characteristics, the photosynthetic pigment contents, the photosynthetic parameters, the photosynthetic enzyme activities and the malate content data were subjected to ANOVA. The statistical analysis was conducted with the R software package. Duncan’s multiple range test was used to compare mean differences among treatments at the 5% probability level.

## Results

### Stoma characteristics

**Figure 1:**
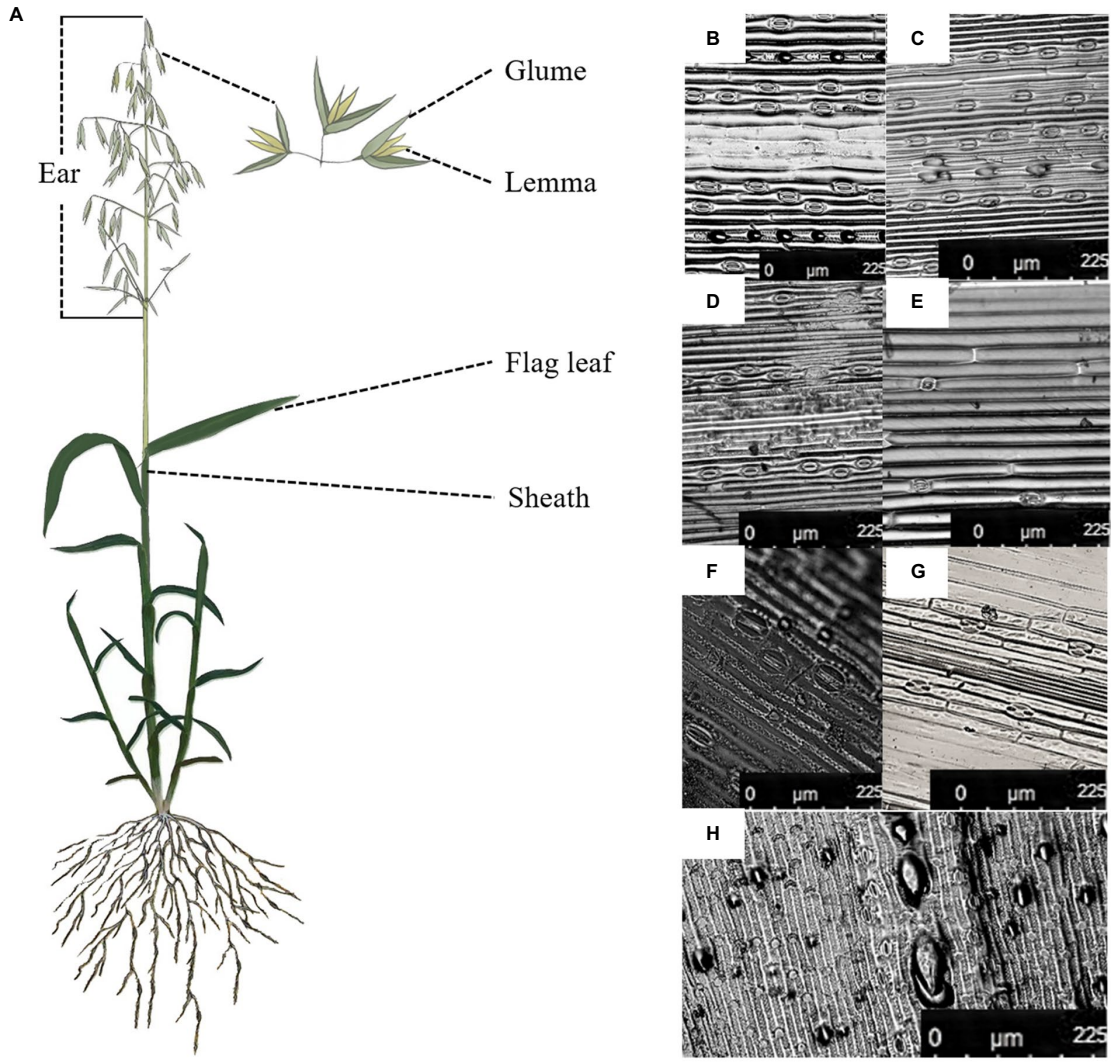
Stoma distribution in different green organs of oat on 7DAA. **(A)**, The flag leaf and the non-leaf organs in the oat plant, **(B)**, the adaxial surface of the flag leaf, **(C)**, the abaxial surface of the flag leaf, **(D)**, the exterior surface of the sheath, **(E)**, the interior surface of the sheath, **(F)**, the exterior surface of the glume, **(G)**, the interior surface of the glume, **(H)**, the exterior surface of the lemma.

Stomata were found on the adaxial and abaxial surface of the flag leaf, the interior and the exterior surface of sheath and the glume and the exterior surface of the lemma in oat (Figure 1). The adaxial surface of the flag leaf had a higher stomatal frequency, stomatal index and guard cell length than of the interior surface of three non-leaf organs (Table 2). Nevertheless, the lower stomatal frequency was found in the abaxial surface of the flag leaf than of the exterior surface of the sheath and the glume, and the exterior surface of the sheath had a higher stomatal index than of the flag leaf.

**Table 2:** Stoma characteristics of the flag leaf, the sheath, the glume and the lemma on 7DAA.

		Stomatal frequency (No. mm^−2^)	Stomatal index	Guard cell length (μm)
Flag leaf	Adaxial surface	90.3a	20.98a	52.39a
	Abaxial surface	47.81c	13.34c	52.76a
Sheath	Interior surface	17.71d	11.35c	37.63c
	Exterior surface	72.60b	16.95b	50.23a
Glume	Interior surface	65.51b	8.03d	33.91d
	Exterior surface	65.51b	10.86c	45.38b
Lemma	Exterior surface	69.05b	12.05c	43.94b

Different small letters within the same column meant a significant difference at the 0.05 probability level.

### Relative water content

On 21DAA and 28DAA, the flag leaf showed a significant decrease in the RWC under drought stress (Table 3). In addition, the RWC was significantly reduced under drought stress in the sheath on 14DAA, 21DAA, and 28DAA, in the glume on 21DAA and 28DAA and the lemma on 21DAA. Nevertheless, the variation of the RWC in the glume and the lemma was lower than in the flag leaf and the sheath.

**Table 3:** The relative water content of the flag leaf, the sheath, the glume and the lemma under drought stress.

		7DAA	14DAA	21DAA	28DAA
Flag leaf	CK	93.95 ± 2.55ab	97.31 ± 1.87a	97.40 ± 0.65a	98.43 ± 0.69a
	D		91.46 ± 5.82ab	88.82 ± 9.19bc	82.38 ± 3.35c
	△		5.85 (6.0%)	8.58 (8.8%)	16.06 (16.3%)
Sheath	CK	85.47 ± 2.24bc	91.09 ± 3.46ab	91.90 ± 0.12a	91.54 ± 1.30a
	D		80.94 ± 4.77c	79.70 ± 2.90c	65.09 ± 5.27d
	△		10.16 (11.1%)	12.20 (13.3%)	26.44 (28.9%)
Glume	CK	93.51 ± 1.67a	94.06 ± 1.56a	94.05 ± 1.36a	92.15 ± 2.05a
	D		91.45 ± 2.14ab	88.67 ± 0.65bc	86.19 ± 2.99c
	△		2.61 (2.8%)	5.38 (5.7%)	5.97 (6.5%)
Lemma	CK	95.43 ± 2.60a	93.45 ± 3.92ab	95.14 ± 0.65a	93.60 ± 0.68ab
	D		94.77 ± 1.46a	90.41 ± 0.96b	93.56 ± 1.66ab
	△		−1.32 (−1.4%)	4.73 (5.0%)	0.04 (0.4%)

Different small letters within the same organ meant a significant difference at the 0.05 probability level.

### Photosynthetic pigment content

**Figure 2:**
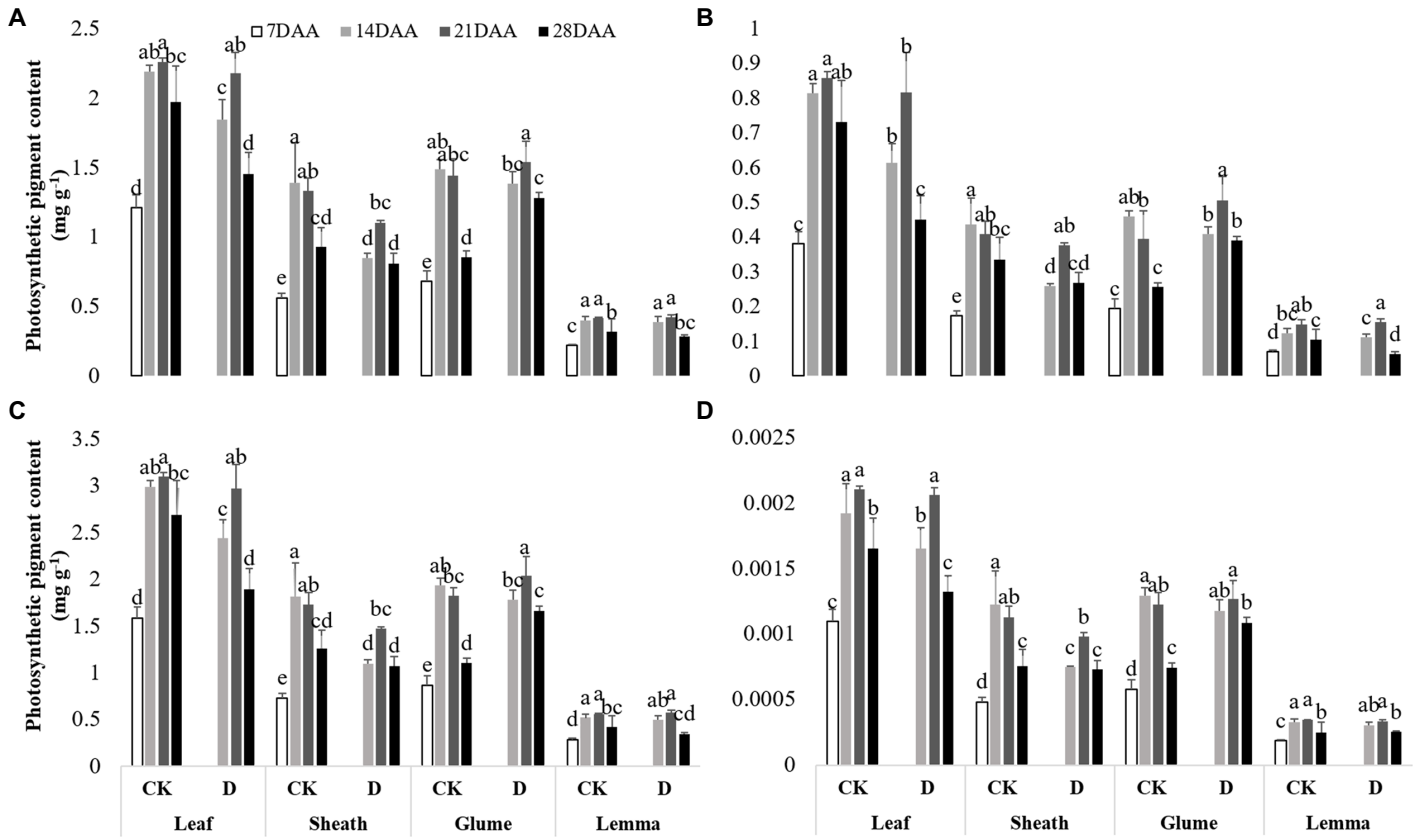
Photosynthetic pigment contents in the flag leaf, the sheath, the glume and the lemma under drought stress. **(A)**, chlorophyll a content (chla), **(B)**, chlorophyll b content (chlb), **(C)**, total chlorophyll content (total chl), **(D)**, xanthophyll content (xan). Different small letters within the same organ meant a significant difference at the 0.05 probability level.

As the growth stage developed, the chla, the chlb, total chl and the xan gradually increased and maximized on 21DAA in the flag leaf under drought stress and CK (Figure 2). In the flag leaf drought stress significantly decreased the chla on 14DAA and 28DAA, the chlb on 14DAA, 21DAA, and 28DAA, total chl on 14DAA and 28DAA and the xan on 14DAA and 28DAA. As the growth stage developed, the chla, the chlb, total chl and the xan gradually increased and maximized on 14DAA under CK and maximized on 21DAA under drought stress in the sheath, the glume and the lemma. Drought stress led to the significant reductions of the chla, the chlb, total chl and the xan on 14DAA in the sheath. The chla on 28DAA, the chlb on 21DAA and 28DAA, total chl on 21DAA and 28DAA and the xan on 28DAA in the glume were increased significantly,while the markedly reduction of chlb was observed in the lemma on 28DAA under drought stress.

### Photosynthetic parameters

As the stage developed, Pn was gradually increased and maximized on 14DAA in the flag leaf (Table 4). The significant decreases of Pn were found in the flag leaf on 14DAA, 21DAA, and 28DAA under water stress. Pn was increased significantly on 21DAA, but was decreased significantly on 28DAA in the sheath. In addition, drought stress led to a marked reduction of Pn in the glume on 14DAA, 21DAA, and 28DAA and in the lemma on 14DAA and 28DAA.

**Table 4:** Photosynthetic parameters in the flag leaf, the sheath, the glume and the lemma under drought stress.

Organs	Treatments	Sampling time	Pn	Gs	NPQ
Flag leaf	CK	7DAA	15.596b	0.248d	1.5855abc
		14DAA	18.569a	0.297c	2.1799ab
		21DAA	18.043a	0.387a	2.5695a
		28DAA	11.446c	0.330b	1.2065bc
	D	14DAA	14.618b	0.197e	2.1237ab
		21DAA	14.425b	0.209e	1.6184abc
		28DAA	8.030d	0.092f	0.8127c
Sheath	CK	7DAA	7.861a	0.148a	3.2851bc
		14DAA	3.561cd	0.048d	3.3337bc
		21DAA	3.384d	0.050d	2.9290c
		28DAA	3.509cd	0.111b	2.7648c
	D	14DAA	3.960c	0.057c	3.8778ab
		21DAA	4.597b	0.045d	4.0806a
		28DAA	2.494e	0.036e	1.9966d
Glume	CK	7DAA	3.459c	0.046c	4.5234ab
		14DAA	4.104b	0.055b	——
		21DAA	7.223a	0.134a	4.4632ab
		28DAA	4.185b	0.055b	3.6021b
	D	14DAA	2.245d	0.028d	5.5363a
		21DAA	4.226b	0.058b	3.3079b
		28DAA	1.241e	0.010e	4.2415ab
Lemma	CK	7DAA	1.786a	0.092a	4.4239a
		14DAA	1.786a	0.052 cd	2.3437b
		21DAA	1.317b	0.068b	3.1157ab
		28DAA	0.196c	0.050d	2.4637b
	D	14DAA	1.444b	0.070b	3.5043ab
		21DAA	1.196b	0.055c	2.5838b
		28DAA	−2.050d	0.039e	3.3998ab

Different small letters within the same organ meant a significant difference at the 0.05 probability level.

Gs was reduced significantly on 14DAA, 21DAA, and 28DAA in the flag leaf. Drought stress significantly increased Gs in the sheath on 14DAA, but decreased on 21DAA, and 28DAA. Gs was decreased significantly in the glume on 14DAA, 21DAA, and 28DAA and in the lemma on 21DAA and 28DAA, but increased on 14DAA in the lemma.

As the stage developed, NPQ was first increased then decreased in the flag leaf under CK, while was decreased under drought stress. NPQ was significantly increased on 21DAA under drought stress, but was decreased on 28DAA in the sheath. Under CK and D treatments, NPQ was higher in sheath, glume and lemma than in flag leaf.

### Photosynthetic enzyme activities

**Figure 3:**
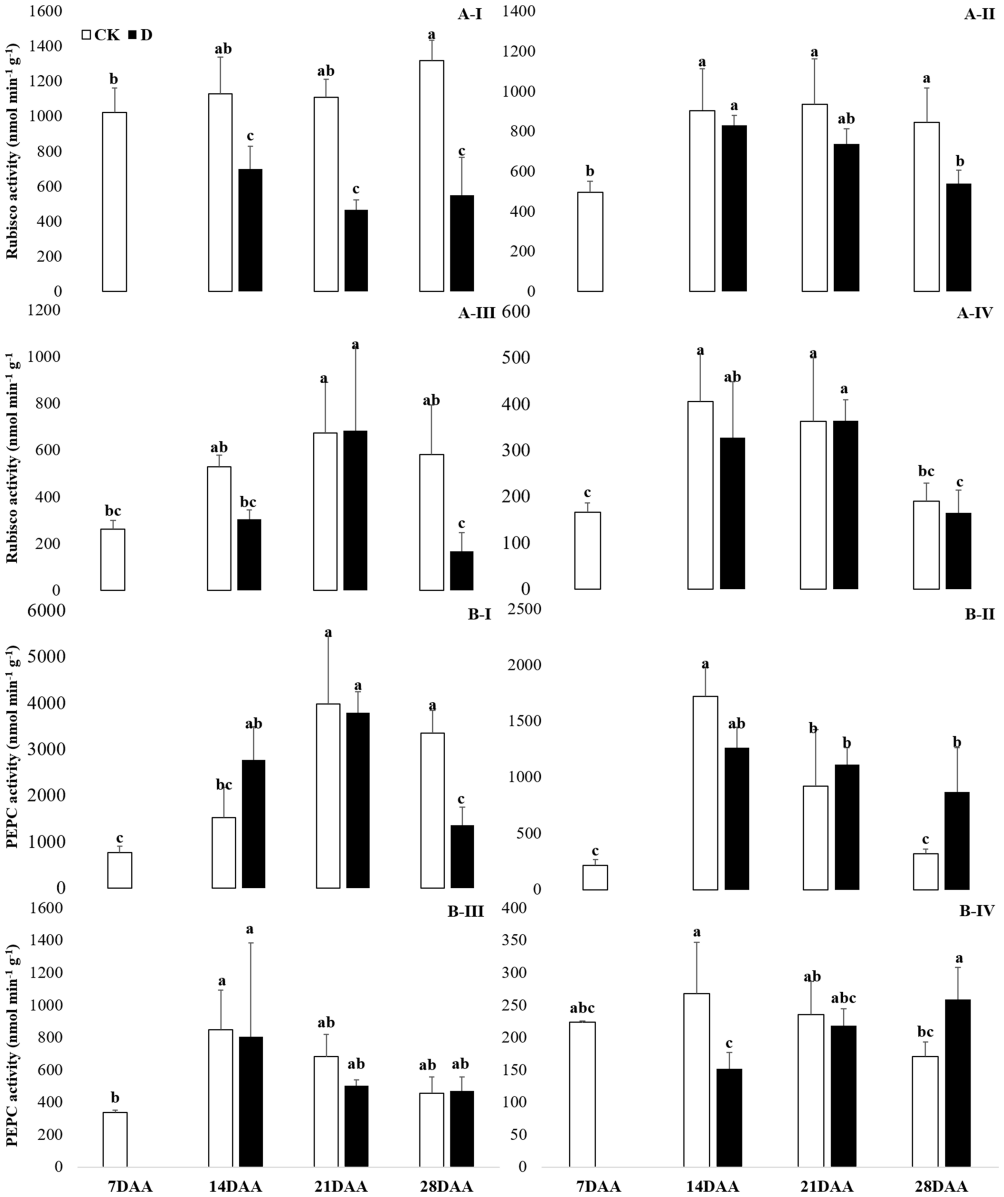
Rubisco **(A)** and PEPC **(B)** activity in the flag leaf **(I)**, the sheath **(II)**, the glume **(III)** and the lemma **(IV)** under drought stress. Different small letters within the same organ meant a significant difference at the 0.05 probability level.

Drought stress significantly decreased the Rubisco activity in the flag leaf on 14DAA, 21DAA, and 28DAA (Figure 3A-I). In addition, Rubisco activity was significantly declined in the sheath and the glume on 28DAA (Figures 3A-II,III).

A significant reduction of PEPC activity was found in the flag leaf on 28DAA under water stress (Figure 3B-I). Drought stress decreased PEPC activity in the lemma on 14DAA (Figure 3B-IV), but increased significantly in the sheath and the lemma on 28DAA (Figures 3B-II,III).

**Figure 4:**
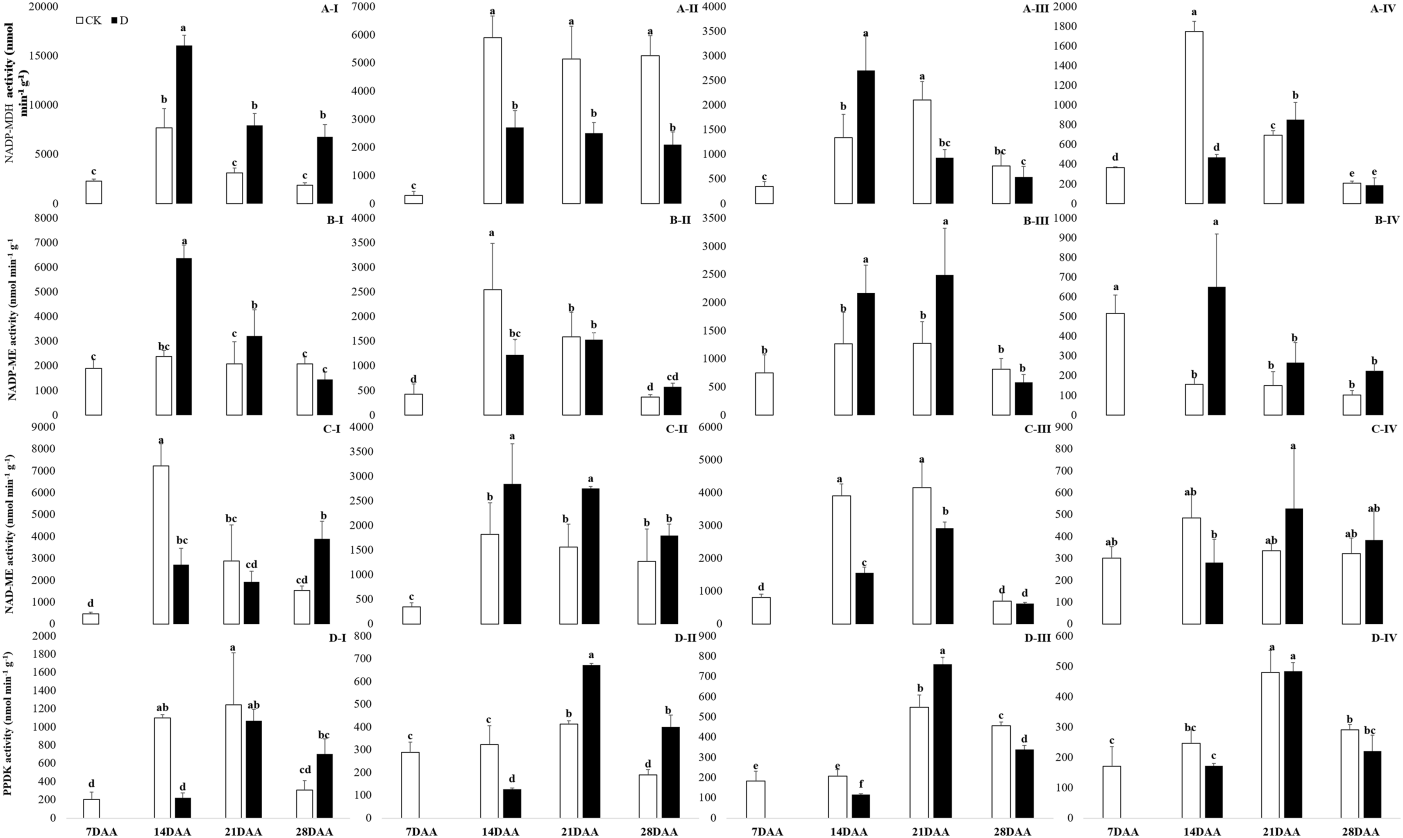
NADP-MDH **(A)**, NADP-ME **(B)**, NAD-ME **(C)** and PPDK **(D)** activity in the flag leaf **(I)**, the sheath **(II)**, the glume **(III)** and the lemma **(IV)** under drought stress. Different small letters within the same organ meant a significant difference at the 0.05 probability level.

Under drought stress, NADP-MDH activity was increased significantly in the flag leaf on 14DAA, 21DAA, and 28DAA (Figure 4A-I). In addition, drought stress led to the markedly decreases of NADP-MDH activity in the sheath on 14DAA, 21DAA, and 28DAA (Figure 4A-II). NADP-MDH activity was significantly increased in the glume on 14DAA and in the lemma on 21DAA (Figure 4A-III), but decreased significantly in the lemma on 14DAA (Figure 4A-IV).

NADP-ME activity was significantly increased in the flag leaf on 14DAA and 21DAA under water stress (Figure 4B-I). Drought stress significantly decreased NADP-ME activity in the sheath on 14 DAA (Figure 4B-II), but increased in the glume on 14DAA and 21DAA (Figure 4B-III) and in the lemma on 14DAA (Figure 4B-IV).

NAD-ME activity was significantly decreased in the flag leaf on 14DAA, but increased on 28DAA under drought stress (Figure 4C-I). Drought stress decreased significantly NAD-ME activity in the glume on 14DAA and 21DAA (Figure 4C-III), but increased significantly in the sheath on 14DAA and 21DAA (Figure 4C-II).

PPDK activity was significantly decreased in the flag leaf on 14DAA under water stress (Figure 4D-I). Drought stress significantly reduced PPDK activity in the sheath on 14DAA (Figure 4D-II) and in the glume on 14DAA and 28DAA (Figure 4D-III), but increased in the sheath and the glume on 21DAA (Figures 4D-II,III).

### Relative expression of *PEPC* and *MDH*

**Figure 5:**
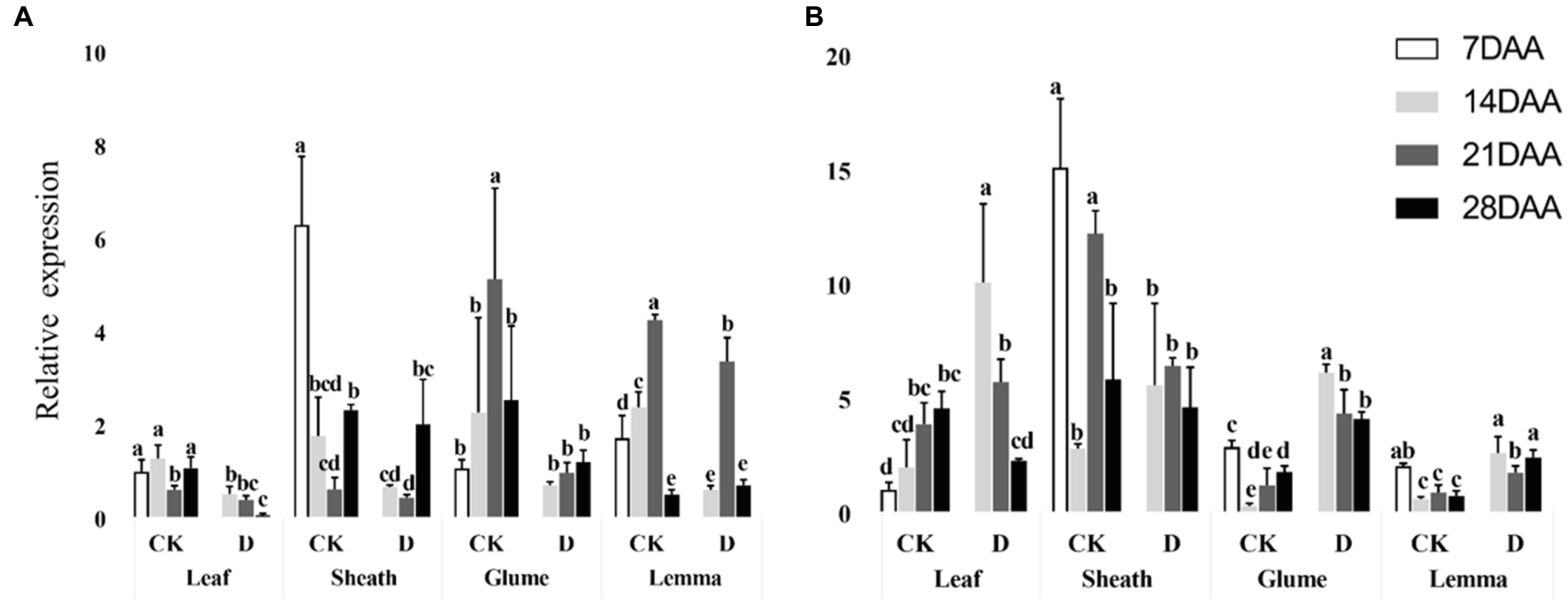
Relative expression of *PEPC* gene **(A)** and *MDH* gene **(B)** in different organs under control and drought stress. Different small letters within the same organ meant a significant difference at the 0.05 probability level.

*PEPC* was significantly downregulated in the flag leaf on 14DAA and 28 DAA under drought stress and was significantly decreased as the stage developed (Figure 5A). In the glume, *PEPC* was significantly downregulated on 14DAA and in the lemma on 14DAA and 21DAA under drought stress (Figure 5A). *MDH* was significantly upregulated in the leaf at 14DAA and significantly down-regulated in the sheath on 21DAA under drought stress (Figure 5B). In addition, MDH was significantly upregulated in the glume and the lemma on 14DAA, 21DAA, and 28DAA under drought stress (Figure 5B). Under drought stress, the sheath, glume and lemma had the higher relative expression of *PEPC* and *MDH* than flag leaf (Figure 5).

### Malate content

**Figure 6:**
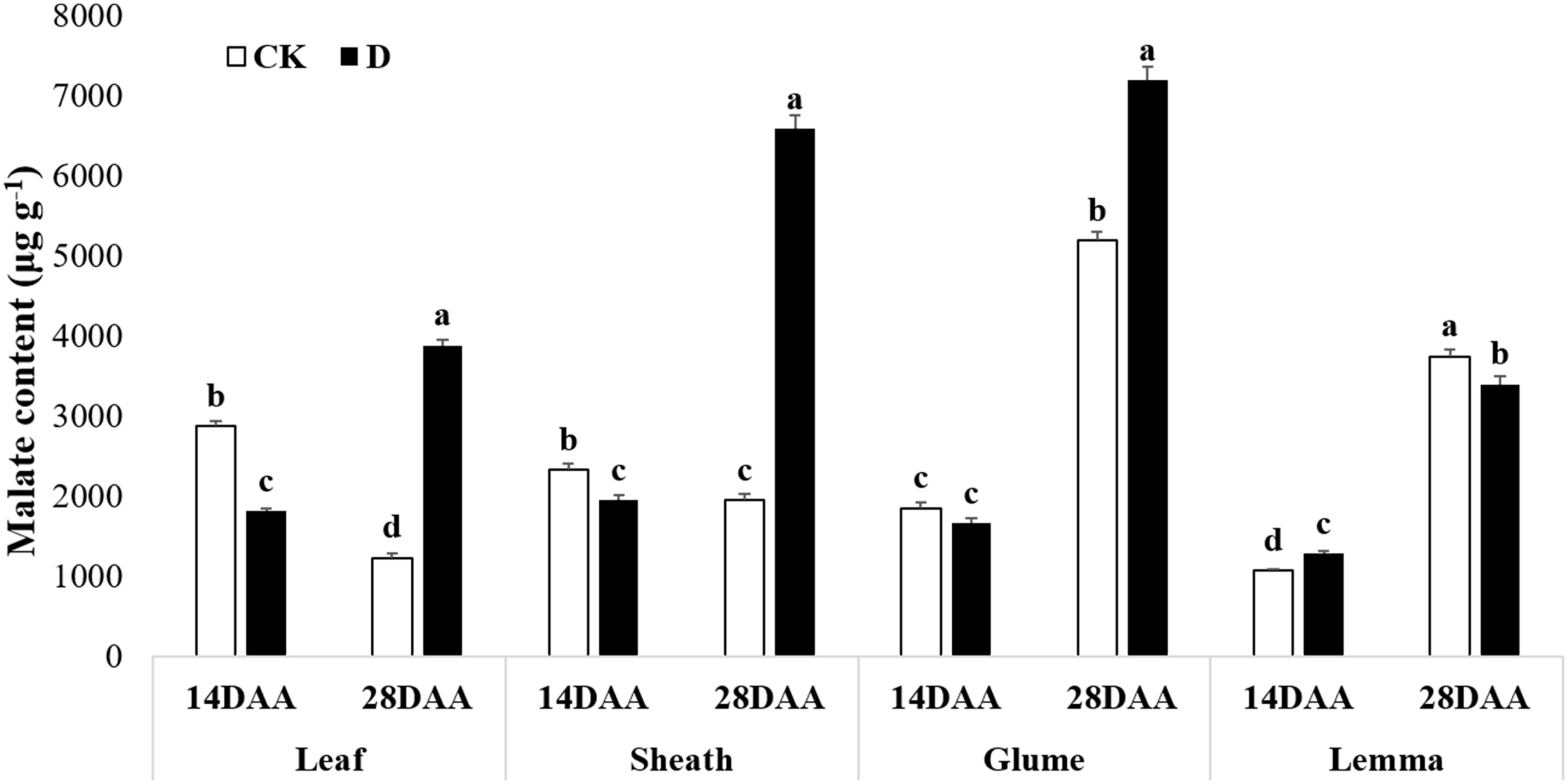
Malate content in the flag leaf, the sheath, the glume and the lemma under drought stress. Different small letters within the same organ meant a significant difference at the 0.05 probability level.

Malate content was significantly decreased in the flag leaf and the sheath on 14DAA under drought stress, but increased significantly in the flag leaf, the sheath and the glume on 28DAA (Figure 6). Drought stress significantly increased malate content in the lemma on 14DAA, but decreased significantly on 28DAA.

## Discussion

### Drought stress affects the relative water content of non-leaf organs

The greater tolerance of non-leaf organs to drought stress has been previously reported in some studies (Xu and Ishii, 1990; Wardlaw, 2002; Tambussi et al., 2005; Jia et al., 2015; Hein et al., 2016). In this study, the glume and the lemma in oat maintained the higher RWC under drought stress with the lower variation compared to the flag leaf (Table 3). The differences in the capacity to maintain high RWC between leaves and non-leaf organs could be due to the variance in sclerophyllous characteristics (Araus et al., 1986) and the capacity of osmotic adjustment (Hein et al., 2016). Compared to leaves, smaller and denser cells, lower intercellular spaces and thicker cellular walls were observed in non-leaf organs including the glume, the lemma and the awn in cereals, which contributed to reducing the damage resulted from drought stress (Li et al., 2006; Tambussi et al., 2007). In addition, drought stress could induce the accumulation of osmotic substances including the proline and the raffinose and increase the osmotic potential in ear organs (Tambussi et al., 2005; Li et al., 2006; Abebe et al., 2010; Jia et al., 2015). The non-leaf organs could ensure better physiological performance by maintaining a buffer function under drought conditions, performing better drought adaptation.

### Response of photosynthetic parameters in non-leaf organs to drought stress

In addition to the differences of RWC, the non-leaf organs and the flag leaf had different changes in the content of photosynthetic pigments and photosynthetic parameters during the drought treatment. Stable photosynthetic performance implies a strong tolerance to drought stress. Chlorophyll content was lower in non-leaf organs than in leaves, which was found in wheat (Lu and Lu, 2004; Lou et al., 2018), cotton (Zhang et al., 2016) and soybean (Andrews and Svec, 1975). However, the greater photosynthetic activity could be measured per chlorophyll in non-leaf organs than in leaves (Andrews and Svec, 1975; Tambussi et al., 2007). In this study, the drought stress could significantly reduce the chla, chlb, total chl, xan and Pn in the flag leaf at three sampling times, while the photosynthetic pigments content in the glume were increased significantly at the late growth stage (21DAA and 28DAA; Figure 2; Table 4). Similarly, the chlorophyll content in non-leaf organs in cotton and wheat was influenced less by the drought condition, compared with leaves (Zhang et al., 2016; Lou et al., 2018). The regulation effect of the xanthophyll cycle to thermal dissipation was verified in rice and oilseed rape (Zhu et al., 2011). In this study, we found that the higher xan was measured in the glume on 28DAA under drought stress (Figure 2D), which reflected the xanthophyll cycle participated in dissipating excess energy in the glume of oat as well under drought conditions. Among the organs evaluated in this study, drought stress significantly reduced the Pn of the flag leaf, the glume and the lemma, not for the sheath, and even the Pn of the lemma on 28DAA was negative (Table 4). Under the drought condition, the green organs would close the stoma to reduce water evaporation, which attenuated dark reaction (Reddy et al., 2004). In addition, persistent drought stress might cause the degradation of photosynthetic pigments, the damage to membrane system and the reduction of synthetase activity (Ladjal et al., 2000; Jaleel et al., 2009). Previous studies reported that the reduction of Pn in bracts was significantly less than in leaves in cotton under drought stress (Wullschleger et al., 1990; Hu et al., 2014; Zhang et al., 2016). The photosynthesis was less sensitive to water deficit in the non-leaf organs than in leaves, showing a larger contribution to the seed yield (Martinez et al., 2003; Jia et al., 2015). NPQ value represents the ability to protect plants from photodamage by dissipating excess light energy under stress conditions. PSII (Photosynthetic system II) in plants was damaged initially during photosynthesis when confronted stress (Xiao et al., 2019). The higher NPQ value was determined in the glume of wheat than in leaves (Kong et al., 2015), which was consistent to our findings that the non-leaf organs had the higher NPQ value. Under drought stress, as the growth stage developed, the NPQ value in the flag leaf decreased, while the non-leaf maintained stable (Table 4). This suggested higher photosynthetic resistance of the non-leaf organs to the water deficit compared with leaves.

### Changes in photosynthetic enzyme activities in non-leaf organs

The results showed that Rubisco activity was markedly influenced by the drought stress in the flag leaf at three growth stages (Figure 3). These findings were in agreement with previous reports for wheat (Guliyev et al., 2008; Simova-Stoilova et al., 2020), rice (Gujjar et al., 2020), cotton (Carmo-Silva et al., 2012; Hu et al., 2014). Rubisco was regarded as the key enzyme of the Calvin cycle (Lawlor et al., 1989), the reduction of Rubisco activity and content resulted in the decline of photosynthetic ability (Hu et al., 2014). In addition to C_3_ pathway enzymes, some C_4_ pathway enzymes were activated in non-leaf organs in C_3_ plants and water deficit could induce the activities (Wei et al., 2003; Jia et al., 2015; Wang et al., 2020). In the present study, PEPC activity was significantly higher in the sheath and the lemma on 28DAA under drought treatment (Figure 3). The drought stress could significantly induce the activities of NADP-MDH, NADP-ME, NAD-ME, and PPDK in non-leaf organs (Figure 4). The previous studies reported that in non-leaf organs, the PEPC activity was significantly higher than in leaves and was induced under drought stress (Imaizumi et al., 1990; Jia et al., 2015; Zhang, 2019; Wang et al., 2020). The PEP in non-leaf organs was considered to have the possibility to recapture the respired CO_2_ in the dark reaction (Singal et al., 1986). Drought stress significantly improved the activities of NADP-MDH, NADP-ME, PPDK and induced the expression of *NADP-MDH-7*, *NADP-ME-1*, *PPDK-1* in the glume of wheat, which was the explanation for the photosynthetic persistence and drought tolerance of non-leaf organs when confronted under water deficit (Jia et al., 2015; Zhang, 2019). In this study, the ear organs had the higher relative expression of *PEPC* and *MDH* than flag leaf under drought stress (Figure 5). Malate is the initial product of photosynthetic CO_2_ fixation in C_4_ plant leaves and ears in wheat as well (Singal et al., 1986). Photosynthetic enzymes in C_4_ cycle played important roles in the anaplerosis of intermediates, such as malate, and supplying carbon skeleton for the amino acids formation (Lea et al., 2001). Therefore, in accordance with the variation of enzyme activities in C_4_ cycle, malate content was significantly increased in the glume and the lemma (Zhang, 2019). The increase of malate content likely contributed to improve the osmotic adjustment and supply carbon skeleton. In this study, drought stress significantly increased the malate content in the lemma on 14DAA and in the sheath and the glume on 28DAA (Figure 6).

## Conclusion

This study has evaluated the changes of the relative water content, the photosynthetic pigment contents, the photosynthetic parameters, the important enzyme activities in C_3_ and C_4_ pathway and the malate content in the flag leaf, the sheath, the glume and the lemma of oat under drought stress. These results suggest that C_4_ photosynthetic enzymes in non-leaf organs, especially in glume, play crucial functions in improving oat’ overall photosynthetic capacity, which contributes to maintaining the stable photosynthetic performance under drought stress conditions.

## Data availability statement

The original contributions presented in the study are included in the article/supplementary material, further inquiries can be directed to the corresponding author.

## Author contributions

HT and HW prepared the initial draft. QZ, WL, JZ, YC, and ZJ revised the manuscript. YS drew the Figure 1. HW designed the experiment. All authors contributed to the article and approved the submitted version.

## Funding

This study was supported by the National Natural Science Foundation of China (32001392), the China Agriculture Research System of MOF and MARA (CARS-34) and “the Fundamental Research Funds for the Central Universities,” Southwest Minzu University (2021PTJS30).

## Conflict of interest

The authors declare that the research was conducted in the absence of any commercial or financial relationships that could be construed as a potential conflict of interest.

## Publisher’s note

All claims expressed in this article are solely those of the authors and do not necessarily represent those of their affiliated organizations, or those of the publisher, the editors and the reviewers. Any product that may be evaluated in this article, or claim that may be made by its manufacturer, is not guaranteed or endorsed by the publisher.
